# The After-History of Gastric Ulcer

**Published:** 1904-02-20

**Authors:** 


### THE AFTER-HISTORY OF GASTRIC ULCER.

Dr. James Russell[1] has collected the histories of 47 cases of gastric ulcer after the patients had left the Birmingham General Hospital, where they were treated during the years 1892–1899. All his cases were in women who had suffered from hæmatemesis, and the length of each case was counted from the first hæmatemesis. One patient or 2·1 per cent. died directly from her gastric disease. Two died from intercurrent maladies. Thirteen or 27·7 per cent. had recovered for periods from two to eight years, without relapse. Seven cases (14·9 per cent) recovered after relapses. These relapses had occurred at periods varying from three or four months to seven years after the first bleeding. In only two of these had hæmatemesis recurred. Four cases are difficult to classify, because in one a gastric operation was performed and in the other three the evidence of recovery is unsatisfactory. Twenty-one patients (44·7 per cent.) still suffer from their complaint, the duration being from two to 13 years. Nine or 10 of these had a second hæmorrhage, and in two cases a third is recorded. Recurrent hæmatemesis while not precluding the possibility of recovery is a serious factor in prognosis pointing at least to a considerable prolongation of the period of active disease. Regarding these cases of non-recovery, Dr. Russell believes that it would be unwise to attempt to draw any definite conclusion as to the number of cases in this group in which surgical interference is advisable. In all probability some of the patients will ultimately recover, whilst it is quite possible that some reports have erred on the side of exaggeration. In others, again, the amount of suffering appears comparatively slight. But on the whole he is inclined to think this group affords some justification for the plea made for more frequent resort to operation in persistent cases of gastric ulcer, always providing the mortality of the operation be kept at a really low figure, and the results are permanently good. At present further evidence on both these points seems to be needed.

[1] Lancet, Jan. 30.

